# Dispatcher instructions for bystander cardiopulmonary resuscitation and neurologically intact survival after bystander-witnessed out-of-hospital cardiac arrests: a nationwide, population-based observational study

**DOI:** 10.1186/s13054-021-03825-w

**Published:** 2021-11-27

**Authors:** Yoshikazu Goto, Akira Funada, Tetsuo Maeda, Yumiko Goto

**Affiliations:** 1 Department of Emergency and Critical Care Medicine, Kanazawa University Hospital, Takaramachi 13-1, Kanazawa, 920-8640 Japan; 2 Department of Cardiology, Osaka Saiseikai Senri Hospital, Tukumodai 1-1-6, Suita, 565-0862 Japan; 3 Department of Cardiology, Yawata Medical Center, Yawata I 12-7, Komatsu, 923-8551 Japan

**Keywords:** Out-of-hospital cardiac arrest, Dispatcher-assisted cardiopulmonary resuscitation, Instruction, Outcome, Epidemiology

## Abstract

**Background:**

The International Liaison Committee on Resuscitation recommends that dispatchers provide instructions to perform compression-only cardiopulmonary resuscitation (CPR) to callers responding to adults with out-of-hospital cardiac arrest (OHCA). This study aimed to determine the optimal dispatcher-assisted CPR (DA-CPR) instructions for OHCA.

**Methods:**

We analysed the records of 24,947 adult patients (aged ≥ 18 years) who received bystander DA-CPR after bystander-witnessed OHCA. Data were obtained from a prospectively recorded Japanese nationwide Utstein-style database for a 2-year period (2016–2017). Patients were divided into compression-only DA-CPR (*n* = 22,778) and conventional DA-CPR (with a compression-to-ventilation ratio of 30:2, *n* = 2169) groups. The primary outcome measure was 1-month neurological intact survival, defined as a cerebral performance category score of 1–2 (CPC 1–2).

**Results:**

The 1-month CPC 1–2 rate was significantly higher in the conventional DA-CPR group than in the compression-only DA-CPR group (before propensity score (PS) matching, 7.5% [162/2169] versus 5.8% [1309/22778], *p* < 0.01; after PS matching, 7.5% (162/2169) versus 5.7% (123/2169), *p* < 0.05). Compared with compression-only DA-CPR, conventional DA-CPR was associated with increased odds of 1-month CPC 1–2 (before PS matching, adjusted odds ratio 1.39, 95% confidence interval [CI] 1.14–1.70, *p* < 0.01; after PS matching, adjusted odds ratio 1.34, 95% CI 1.00–1.79, *p* < 0.05).

**Conclusion:**

Within the limitations of this retrospective observational study, conventional DA-CPR with a compression-to-ventilation ratio of 30:2 was preferable to compression-only DA-CPR as an optimal DA-CPR instruction for coaching callers to perform bystander CPR for adult patients with bystander-witnessed OHCAs.

**Supplementary Information:**

The online version contains supplementary material available at 10.1186/s13054-021-03825-w.

## Background

Bystander cardiopulmonary resuscitation (CPR) before the arrival of the emergency medical service (EMS) is associated with an increased chance of survival after out-of-hospital cardiac arrest (OHCA) between two- and threefold higher compared with no bystander CPR [1, 2]. The incidence rate of bystander CPR for OHCA varies from 26 to 86% [2–4]. Dispatcher-assisted CPR (DA-CPR) has been implemented to increase the performance of bystander CPR and ultimately improve survival. A recent systematic review showed that DA-CPR is associated with improved outcomes compared with no bystander CPR in terms of survival with favourable neurological outcomes, survival to hospital discharge, and return of spontaneous circulation [5]. Based on this evidence, the International Liaison Committee on Resuscitation (ILCOR) strongly recommends that dispatchers provide instructions to perform compression-only CPR without rescue breaths to callers for adults with suspected OHCA as a minimum if bystanders are untrained or unskilled in CPR [6]. ILCOR places a higher value on the initiation of bystander compressions and a low value on the possible harm of delayed ventilation. The 2021 guidelines of the European Resuscitation Council (ERC) are consistent with those of the ILCOR and recommend that callers perform compression-only CPR for adults with suspected OHCA [7]. To date, there have been three randomised controlled trials (RCT) comparing compression-only CPR with conventional CPR (compression with rescue breaths) after instructions from EMS dispatchers to untrained bystanders [8–10]. All these trials concluded that the overall survival rate was similar between compression-only CPR and conventional CPR groups. However, these trials used a compression-to-ventilation ratio of 15:2 as a conventional CPR instruction and excluded patients with hypoxic arrest from the analyses. Since 2005, the CPR guidelines have recommended a compression-to-ventilation ratio of 30:2 as conventional CPR [11, 12]. The optimal DA-CPR instructions for callers to perform bystander CPR for patients with OHCA before EMS arrival in the current era of 30:2 compression-to-ventilation ratio have not been fully investigated.

In this study, we aimed to determine the optimal dispatcher-assisted CPR instructions for bystanders to perform CPR after bystander-witnessed OHCA with all causes using the Japanese nationwide registry data.

## Methods

### Study design and setting

This nationwide, population-based observational study included all adult patients (aged ≥ 18 years) who experienced OHCA and were resuscitated by EMS personnel in Japan between 1 January 2016 and 31 December 2017. Patients were excluded based the following criteria: (1) aged < 18 years; (2) witnessed by EMS personnel; (3) did not receive resuscitation from EMS personnel or bystanders; (4) unwitnessed arrest; (5) received rescue breathing-only bystander CPR; and (6) had unknown outcomes or age or some time variables.

In Japan, the nationwide EMS system is supervised by the Fire and Disaster Management Agency (FDMA), while local fire stations operate the local EMS systems. As of 2017, Japan has 732 fire departments and 5140 ambulance teams [13]. During the study period, all EMS personnel performed CPR according to the Japanese guidelines [14]. Moreover, emergency lifesaving technicians who were EMS personnel used several other resuscitation techniques, including automated external defibrillators, airway adjuncts, peripheral intravenous catheters, and administration of Ringer’s lactate solution [13]. In the field, only specially trained emergency lifesaving technicians, upon receiving instructions from an online physician, are permitted to insert a tracheal tube and administer intravenous adrenaline (epinephrine). EMS personnel in Japan are legally prohibited from terminating resuscitation in the field. Therefore, most OHCA patients receive CPR from EMS personnel before being transported to a hospital.

In Japan, since 2000, emergency dispatch centres have become increasingly active in relaying CPR instructions to bystanders who can then perform conventional CPR [15–17]. In 2006, DA-CPR instructions were revised from conventional DA-CPR to compression-only DA-CPR. Since then, emergency telephone dispatchers in Japan are required to provide instructions on how to perform compression-only DA-CPR if it is challenging for bystanders to administer rescue breaths. Dispatchers can offer CPR instructions for either conventional DA-CPR or compression-only DA-CPR to callers.

### Data collection and quality control

In 2005, the FDMA launched an ongoing, prospective, population-based observational study involving patients with OHCA who had received resuscitation from EMS personnel in Japan [13]. Since 2005, with the cooperation of the physician-in-charge, EMS personnel from each centre recorded the data of patients with OHCA using an Utstein-style template [18]. These data were transferred to local fire stations and subsequently integrated into the registry on the FDMA database server. All data were transferred and stored in the nationwide database developed by the FDMA for public use. The FDMA permitted access to the database and provided anonymous data for our analysis. The main variables included in the dataset were sex, age, aetiology of arrest, initially identified cardiac rhythm, bystander-witnessed status, type of witness, type of bystander CPR, time of collapse recognition, time of emergency call, time of vehicle arrival at the scene, time of CPR initiation by EMS, 1-month survival, and neurological outcomes 1 month after cardiac arrest. The aetiology of arrest was presumed to be cardiac unless evidence suggested noncardiac medical causes, traumatic cause, submersion, drug overdose, drowning, electrocution, asphyxia, or any other noncardiac causes. The physicians-in-charge and EMS personnel attempted to determine the origin of the arrest. Neurological outcomes were defined using the Cerebral Performance Category (CPC) scale (category 1, good cerebral performance; category 2, moderate cerebral disability; category 3, severe cerebral disability; category 4, coma or vegetative state; and category 5, death) [18]. CPC categorisation was determined by the physician-in-charge 1 month after cardiac arrest. Information on bystander interventions and dispatchers providing CPR instructions was obtained by EMS personnel who interviewed the bystanders before leaving the scene. All data were electronically recorded by EMS personnel and/or the EMS centre.

### Study endpoints

The primary outcome measure was 1-month neurologically intact survival, defined as a CPC score of 1 or 2. The secondary outcome measure was the 1-month survival after OHCA.

### Statistical analysis

We compared 1-month outcomes after OHCA between the conventional DA-CPR and compression-only DA-CPR groups. To perform rigorous adjustment for differences in the baseline characteristics of patients, we utilised both propensity-score matching analyses and multivariable logistic regression analyses before matching or conditional logistic regression analysis after matching to adjust for selection bias when comparing outcomes between the two groups.

In propensity score (PS) matching analyses, we estimated two propensity scores by fitting a logistic regression model that included seven variables based on previous literatures [1, 19–22]: calendar year, sex, age, Japanese regions (rural or urban), witnessed status (family members or non-family members), causes (presumed cardiac or non-cardiac), and call-to-response time (time from emergency call receipt to EMS arrival at the patient’s location). We performed one-to-one nearest neighbour matching between patients who received conventional DA-CPR and those who received compression-only DA-CPR without replacement, using a calliper width equal to 0.20 of the standard deviation of the logit of the propensity score [23]. Before analysing outcomes, we assessed the success of the propensity matching procedure by comparing the distribution of patient characteristics in the matched sample by calculating an absolute standardised difference [24]. An absolute standardised difference of ≥ 0.1 was considered indicative of a significant difference between the two cohorts [25]. To compare the outcomes between the two types of DA-CPR, we utilised either the Chi-square test or Fisher’s exact test in the pre-propensity-matched patients and McNemar’s test in post-propensity-matched patients.

In the multivariable logistic regression analysis before matching or conditional logistic regression analysis after matching, 13 potential prehospital confounders for the analytical model were selected based on biological plausibility and data from previous studies which were as follows: calendar year, sex, age, Japanese regions (rural or urban), witnessed status (family members or non-family members), initial cardiac rhythm, type of DA-CPR, advanced airway management, adrenaline administration, causes (cardiac or non-cardiac), call-to-response time, collapse-to-initiation of bystander CPR time, and bystander CPR duration.

Continuous variables are expressed as means and standard deviations, whereas categorical variables are expressed as percentages. As an estimate of effect size and variability, we reported odds ratios (ORs) with 95% confidence intervals (CI). We used the Kruskal–Wallis test followed by Dunn’s post hoc test to analyse continuous variables. The Chi-square test and univariate logistic regression analysis were performed to compare the characteristics and outcomes of the categorical variables. All statistical analyses were performed using the JMP statistical package, version 15.2-Pro (SAS Institute Inc., Cary, NC, USA). All reported tests were two-tailed, and a *p* value of < 0.05 was considered statistically significant.

## Results

**Fig. 1:**
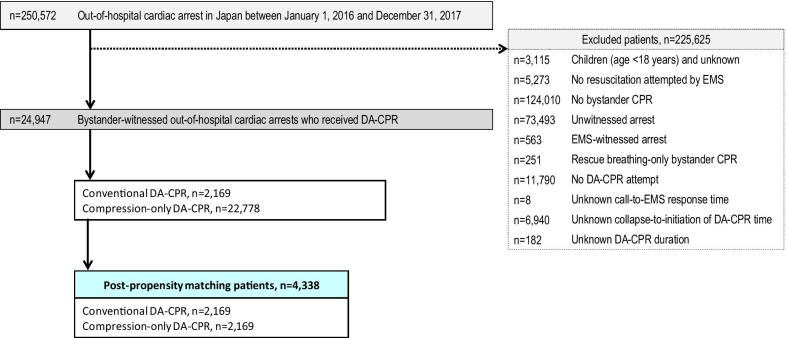
Flowchart of the patient inclusion criteria. CPR cardiopulmonary resuscitation; DA dispatcher-assisted; EMS emergency medical services

Details of attempted resuscitations performed for 250,572 OHCA patients between 2016 and 2017 are documented in the database. Figure 1 presents the inclusion and exclusion criteria of the study. Ultimately, 24,947 patients (10.0% of registered patients) who experienced bystander-witnessed OHCA and received DA-CPR were eligible for analysis. Based on the type of DA-CPR, we divided OHCA patients into conventional DA-CPR (*n* = 2169) and compression-only DA-CPR (*n* = 22,778). Patient matching was achieved for 2169 of the 2169 patients who received conventional DA-CPR (100%) and 2169 of the 22,778 patients with compression-only DA-CPR (9.5%). The baseline characteristics of pre- and post-PS matching are shown in Table 1. Before PS matching, there was a substantial imbalance in the proportions of males, witnessed by family members, collapse-to-initiation of DA-CPR time, and duration of DA-CPR between the two groups. After PS matching, the absolute standardised differences in the matched cohorts improved considerably.

**Table 1:** Baseline characteristics of unmatched and matched patients

Characteristic	Pre-propensity score matching			Post-propensity score matching		
	Conventional DA-CPR	Compression-only DA-CPR	ASD^a^	Conventional DA-CPR	Compression-only DA-CPR	ASD^a^
	(*n* = 2169)	(*n* = 22,778)		(*n* = 2169)	(*n* = 2169)	
Year						
2016	1070 (49.3)	11,065 (48.6)	0.02	1070 (49.3)	1084 (50.0)	0.01
2017	1099 (50.7)	11,713 (51.4)	0.02	1099 (50.7)	1085 (50.0)	0.01
Sex, male	1151 (53.1)	13,416 (58.9)	0.12	1151 (53.1)	1154 (53.2)	0.00
Age, y, mean (SD)	76.7 (15.7)	76.5 (14.8)	0.01	76.7 (15.7)	77.2 (14.9)	0.04
Rural regions^b^	507 (23.4)	5649 (24.8)	0.03	507 (23.4)	424 (19.6)	0.09
Witnessed by family member	944 (43.5)	15,028 (66.0)	0.46	944 (43.5)	951 (43.9)	0.01
Presumed cardiac cause	1292 (59.6)	13,783 (60.5)	0.02	1292 (59.6)	1309 (60.4)	0.02
Call-to-response time, min, mean (SD)	10.1 (4.3)	9.5 (3.9)	0.14	10.1 (4.3)	10.1 (4.2)	0.01
Initial shockable rhythm	304 (14.0)	3463 (15.2)	0.03	304 (14.0)	297 (13.7)	0.01
Advanced airway management	1052 (48.5)	10,530 (46.2)	0.05	1052 (48.5)	979 (45.1)	0.07
Epinephrine administration	702 (32.4)	7565 (33.2)	0.02	702 (32.4)	711 (32.8)	0.01
Collapse-to-initiation of DA-CPR time, min, mean (SD)	3.7 (5.2)	4.7 (5.9)	0.17	3.7 (5.2)	4.2 (5.6)	0.09
Duration of DA-CPR, min, mean (SD)	8.1 (4.0)	7.5 (3.7)	0.17	8.1 (4.0)	7.9 (4.0)	0.05

Values are reported as *n* (%) unless indicated otherwise

ASD absolute standardsed difference; DA-CPR dispatcher-assisted cardiopulmonary resuscitation; PS propensity score; SD standard deviation

^a^An ASD of equal or more than 0.1 was considered to indicate a substantial imbalance between the two groups

^b^The rural area constituted 19 prefectures with a population of less than 200 inhabitants per km^2^

**Fig. 2:**
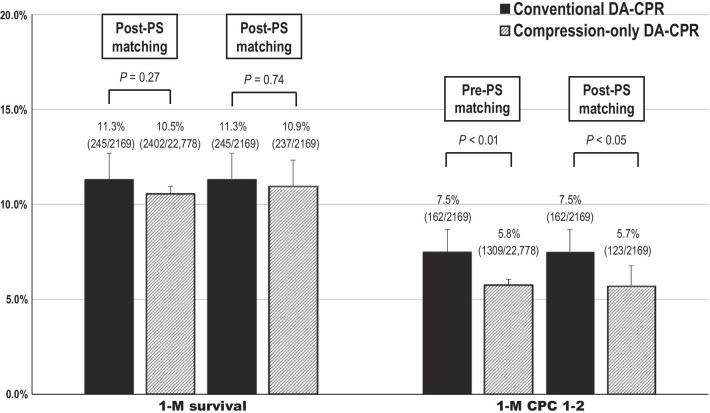
Unadjusted 1-month outcomes pre- and post-propensity score matching. CPC cerebral performance category; DA-CPR dispatcher-assisted cardiopulmonary resuscitation; PS propensity score

**Fig. 3:**
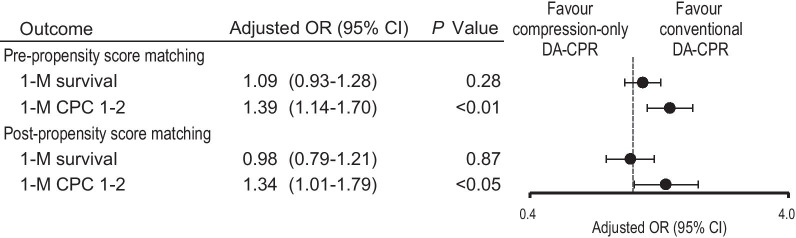
Adjusted odds ratios of conventional DA-CPR for 1-month outcomes. CI confidence interval; CPC cerebral performance category; DA-CPR dispatcher-assisted cardiopulmonary resuscitation; OR odds ratio

The results of unadjusted 1-month outcome comparisons between the two groups before and after PS matching are shown in Fig. 2. There was no significant difference between the two groups in the unadjusted rates of 1-month survival, regardless of PS matching. The rates of 1-month CPC 1–2 were significantly higher in the conventional DA-CPR group than in the compression-only DA-CPR group before and after PS matching (pre-PS matching, 7.5% vs. 5.8%, *p* < 0.01; post-PS matching, 7.5% vs. 5.7%, *p* < 0.05). Relative risks of conventional DA-CPR for 1-month CPC 1–2 were 1.30 (95% CI 1.12–1.51) before PS matching and 1.31 (1.23–1.51) after PS matching. A multivariable logistic regression model revealed that conventional DA-CPR was associated with increased odds of 1-month CPC 1–2 compared with compression-only DA-CPR regardless of PS matching (Fig. 3). However, there was no significant difference in the adjusted OR for 1-month survival between the two groups, regardless of PS matching. Results of subgroup analyses are reported in Additional files 1 (Figure S1) and 2 (Figure S2).

## Discussion

This nationwide, population-based observational study in Japan demonstrates that compared with compression-only DA-CPR, conventional DA-CPR with a compression-to-ventilation ratio of 30:2 was associated with a 31.7% relative increase in 1-month neurologically intact survival, but not in 1-month survival after PS matching. To the best of our knowledge, this is the first and largest cohort study to show a better association between conventional DA-CPR and favourable neurological outcomes after OHCA compared with compression-only DA-CPR.

To increase the incidence rate of bystander CPR, compression-only CPR by bystanders has been widely accepted due to its simpler form of resuscitation with reduced barriers to transmission of infectious diseases [26–28]. The implementation of DA-CPR programs has been found to be associated with increased bystander CPR incidence rate and survival with favourable functional outcome [29]. Previous RCTs [8–10], which used a compression-to-ventilation ratio of 15:2 as a conventional DA-CPR, showed a neutral result in survival to hospital discharge or 30 days after OHCA between compression-only and conventional DA-CPR. The results of the present study, which used a compression-to-ventilation ratio of 30:2 as a comparator, were consistent with those of the RCTs in comparison of 1-month survival between compression-only and conventional DA-CPRs. However, conventional DA-CPR was found to be associated with increased odds of the 1-month CPC 1–2 rate compared with compression-only DA-CPR. Although deleterious effects on haemodynamics during CPR have been shown in a swine model of conventional CPR with a 30:2 compression-to-ventilation ratio compared with compression-only CPR due to interruptions of chest compression during the rescue breaths [30], the superiority of conventional DA-CPR with a compression-to-ventilation ratio of 30:2 for favourable neurological outcomes in the present study implies that oxygen supply may be more important for brain function than hemodynamic sustainability during CPR in clinical settings.

One concern in performing conventional DA-CPR is the time delay in initiating bystander CPR compared with compression-only DA-CPR when bystanders have not been trained in CPR before. In the present study, contrary to expectation, collapse-to-initiation of DA-CPR time was shorter in the conventional DA-CPR than in the compression-only DA-CPR before PS matching by approximately 1 min (mean time, 3.7 min vs. 4.7 min, Table 1). This implies that the proportion of trained bystanders may be higher in the conventional DA-CPR group than in the compression-only DA-CPR group. However, there are no data on whether bystanders have experienced CPR training before or not in the present study. A previous study showed that previously trained bystanders who received the same telephonic instructions performed high-quality CPR with rescue breaths better than those who received telephonic instructions without prior CPR training [31]. In addition, their willingness to perform bystander CPR and their ability to integrate knowledge and psychomotor skills may be higher in the conventional DA-CPR group than in the compression-only DA-CPR group. As aforementioned, the three previous RCTs [8–10] compared compression-only DA-CPR with conventional DA-CPR involving bystanders without previous CPR training. However, these RCTs did not reveal a comparison of neurological outcomes between conventional DA-CPR and compression-only DA-CPR. As of 2021, the national Swedish TANGO2 trial (clin trials, NCT03981107) is now underway to investigate whether EMS instructions to perform a compression-only CPR to bystanders with prior CPR training is non-inferior or better in 30-day survival and neurological outcome than conventional CPR with a compression-to-ventilations ratio of 30:2 in bystander-witnessed OHCA [32]. Until the accumulation of research on conventional DA-CPR with a compression-to-ventilations ratio of 30:2 versus compression-only DA-CPR and outcomes, conventional DA-CPR should be provided by trained bystanders and compression-only DA-CPR should be provided by untrained bystanders or trained bystanders unwilling to provide rescue breaths along with the current guidelines for basic life support [6, 7].

One of the potential reasons why conventional DA-CPR has greater benefits than compression-only DA-CPR in neurologically intact survival may be explained by the better quality of bystander CPR. Earlier studies showed that the quality of chest compression decreased more rapidly in groups with compression-only CPR than in those with conventional CPR due to increased physical fatigue [33, 34]. Another study also showed that conventional CPR had significantly more adequate depth of chest compressions than the compression-only CPR 1 min after starting CPR [35]. Compression-only CPR might cause rescuers with less muscle strength, low body weight, or older age to become fatigued quickly [36]. However, we had no data regarding bystander CPR quality and bystanders’ physical information, including their age.

The results of the present investigation are inconsistent with those from a study by Kitamura [27], which analysed bystander-witnessed OHCA of medical origin using an identical Japanese database between 2005 and 2014. In their report, approximately 50% of patients received dispatcher assistance in both conventional and compression-only CPR groups. They showed that the compression-only CPR group had a more favourable neurological outcome than the conventional CPR group after PS matching (adjusted OR 1.14; 95% CI 1.09–1.22). However, they did not include EMS-response time and bystander CPR duration as confounding factors, which are key factors associated with outcome, in calculating adjusted ORs for favourable neurological outcomes. In the present study, adjusted OR of conventional DA-CPR was calculated including those time variables as one of the confounders and was found to be superior to compression-only DA-CPR with adjusted OR of 1.34 (95%, CI 1.01–1.79).

This study has certain limitations in addition to the aforementioned. First, there may be some differences in dispatcher-assisted CPR protocols among the local fire departments because the FDMA in Japan has provided a standard protocol for dispatcher-assisted CPR instructions and recommended the modification of its content according to liaison with the local medical control area. Second, the actual aetiology of cardiac arrest has not been completely verified. Therefore, we could not analyse the association between the two types of DA-CPR and outcomes stratified by hypoxic origin. Third, although we used a uniform data collection procedure, a large sample size, propensity score matching analyses, and a population-based design, we cannot exclude the possibility of uncontrolled confounders, such as pre-existing comorbidities, location of the arrest, quality of bystander CPR, and in-hospital treatments because the study was retrospective and observational. Therefore, we could not include these data in our analyses. Fourth, as with all epidemiological studies, selection bias may have occurred, and the data may have lacked integrity and validity. Finally, the relevance of our results to other communities with different emergency care systems and protocols remains unknown.

## Conclusions

Within the limitations of this retrospective observational study, conventional DA-CPR with a compression-to-ventilation ratio of 30:2 was preferable to compression-only DA-CPR as an optimal CPR instruction for coaching callers to perform bystander CPR for adult bystander-witnessed OHCAs.

## Supplementary Information

**Additional file 1**. **Figure S1**. Subgroup analysis for adjusted odds ratios of conventional DA-CPR for survival rate. CI, confidence interval; DA-CPR, dispatcher-assisted cardiopulmonary resuscitation; OR, odds ratio. A total of 22 subgroup analyses of adjusted ORs of conventional DA-CPR for 1-month survival after propensity score matching compared with compression-only DA-CPR are shown. There were no significant differences in the 1-month survival rate between the two groups.

**Additional file 2**. **Figure S2**. Subgroup analysis for adjusted odds ratios of conventional CPR for CPC 1-2 rate. CI, confidence interval; CPC, cerebral performance category; DA-CPR, dispatcher-assisted cardiopulmonary resuscitation; OR, odds ratio. A total of 22 subgroup analyses of adjusted ORs of conventional DA-CPR for 1-month CPC 1-2 after propensity score matching compared with compression-only DA-CPR are shown. Most subgroup analyses for 1-month CPC 1-2 rate revealed no significant differences between the two groups. However, conventional DA-CPR was associated with increased odds of 1-month CPC 1–2 rate in six subgroup analyses: urban area, age ≥65 years, initial non-shockable rhythm, advanced airway management, presumed cardiac cause, and call-to-response time (5–9 min).

## Data Availability

The datasets generated and/or analysed during the current study are not publicly available because of the Fire and Disaster Management Agency (FDMA) regulations, but are available from the corresponding author upon reasonable request.

## Abbreviations

CI: Confidence interval
CPC: Cerebral Performance Category
CPR: Cardiopulmonary resuscitation
DA-CPR: Dispatcher-assisted cardiopulmonary resuscitation
EMS: Emergency medical service
ERC: European Resuscitation Council
FDMA: Fire and Disaster Management Agency
ILCOR: International Liaison Committee on Resuscitation
OHCA: Out-of-hospital cardiac arrest
OR: Odds ratio
PS: Propensity score
RCT: Randomised controlled trial

## References

[1] Sasson C, Rogers MAM, Dahl J, Kellerman AL (2010). Predictors of survival from out-of-hospital cardiac arrest: a systematic review and meta-analysis. Cir Cardiovasc Qual Outcomes.

[2] Wissenberg M, Lippert FK, Folke F, Weeke P, Hansen CM, Christensen EF (2013). Association of national initiatives to improve cardiac arrest management with rates of bystander intervention and patient survival after out-of-hospital cardiac arrest. JAMA.

[3] Nichol G, Leroux B, Wang H, Callaway CW, Sopko G, Weisfeldt M (2015). Trial of continuous or interrupted chest compressions during CPR. N Eng J Med.

[4] Kiguchi T, Okubob M, Nishiyama C, Maconochie I, Ong MEH, Kerng KB (2020). Out-of-hospital cardiac arrest across the world: first report from the International Liaison Committee on Resuscitation (ILCOR). Resuscitation.

[5] Nikolaou N, Dainty KN, Couper K, Morley P, Tijssen J, Vaillancourt C (2019). A systematic review and meta-analysis of the effect of dispatcher-assisted CPR on outcomes from sudden cardiac arrest in adults and children. Resuscitation.

[6] Olasveengen TM, de Caen AR, Mancini ME, Maconochie IK, Aickin R, Atkins DL (2017). 2017 international consensus on cardiopulmonary resuscitation and emergency cardiovascular care science with treatment recommendations summary. Resuscitation.

[7] Semeraro F, Greif R, Böttiger BW, Burkart R, Cimpoesu D, Georgiou M (2021). European resuscitation council guidelines 2021: systems saving lives. Resuscitation.

[8] Hallstrom AP (2000). Dispatcher-assisted "phone" cardiopulmonary resuscitation by chest compression alone or with mouth-to-mouth ventilation. Crit Care Med.

[9] Rea TD, Fahrenbruch C, Culley L, Donohoe RT, Hambly C, Innes J (2010). CPR with chest compression alone or with rescue breathing. N Eng J Med.

[10] Svensson L, Bohm K, Castrèn M, Pettersson H, Engerström L, Herlitz J (2010). Compression-only CPR or standard CPR in out-of-hospital cardiac arrest. N Eng J Med.

[11] Hazinski MF, Nadkarni VM, Hickey RW, O'Connor R, Becker LB, Zaritsky A (2005). Major changes in the 2005 AHA Guidelines for CPR and ECC: reaching the tipping point for change. Circulation.

[12] Sandroni C, Cavallaro F (2008). The 2005 European Guidelines for cardiopulmonary resuscitation: major changes and rationale. Minerva Anestesiol.

[13] Ambulance Service Planning Office of Fire and Disaster Management Agency of Japan. Effect of first aid for cardiopulmonary arrest (**in Japanese**). https://www.fdma.go.jp/publication/rescue/items/kkkg_h29_01_kyukyu.pdf. Accessed 2 June 2021.

[14] Japan Resuscitation Council CPR Guidelines Committee. 2015 Japanese Guidelines for Emergency Care and Cardiopulmonary Resuscitation. Tokyo: Igaku shoin; 2016 (**in Japanese**).

[15] Goto Y, Maeda T, Goto Y (2014). Impact of dispatcher-assisted bystander cardiopulmonary resuscitation on neurological outcomes in children with out-of-hospital cardiac arrests: a prospective, nationwide, population-based cohort study. J Am Heart Assoc.

[16] Goto Y, Funada A, Goto Y (2018). Conventional versus chest-compression-only cardiopulmonary resuscitation by bystanders for children with out-of-hospital cardiac arrest. Resuscitation.

[17] Goto Y, Funada A, Maeda T, Goto Y (2021). Temporal trends in neurologically intact survival after paediatric bystander-witnessed out-of-hospital cardiac arrest: a nationwide population-based observational study. Resusc Plus..

[18] Perkins GD, Jacobs IG, Nadkarni VM, Bhanji F, Bossaert LL, Chamberlain D (2015). Cardiac arrest and cardiopulmonary resuscitation outcome reports: update of the Utstein Resuscitation Registry Templates for Out-of-Hospital Cardiac Arrest: a statement for healthcare professionals from a task force of the International Liaison Committee on Resuscitation (American Heart Association, European Resuscitation Council, Australian and New Zealand Council on Resuscitation, Heart and Stroke Foundation of Canada, InterAmerican Heart Foundation, Resuscitation Council of Southern Africa, Resuscitation Council of Asia); and the American Heart Association Emergency Cardiovascular Care Committee and the Council on Cardiopulmonary, Critical Care. Perioper Resusc Circ.

[19] Goto Y, Maeda T, Nakatsu-Goto Y (2013). Neurological outcomes in patients transported to hospital without a prehospital return of spontaneous circulation after cardiac arrest. Crit Care.

[20] Goto Y, Funada A, Goto Y (2018). Relationship between emergency medical services response time and bystander intervention in patients with out-of-hospital cardiac arrest. J Am Heart Assoc.

[21] Goto Y, Funada A, Maeda T, Okada H, Goto Y (2019). Sex-specific differences in survival after out-of-hospital cardiac arrest: a nationwide, population-based observational study. Crit Care.

[22] Goto Y, Funada A, Maeda T, Goto Y (2021). Association of dispatcher-assisted cardiopulmonary resuscitation with initial shockable rhythm and survival after out-of-hospital cardiac arrest. Eur J Emerg Med.

[23] Rosenbaum PR, Donald BR (1985). Constructing a control group using multivariate matched sampling methods that incorporate the propensity score. Am Stat.

[24] Austin PC (2011). An introduction to propensity score methods for reducing the effects of confounding in observational studies. Multivariate Behav Res.

[25] Normand ST, Landrum MB, Guadagnoli E, Ayanian JZ, Ryan TJ, Cleary PD (2001). Validating recommendations for coronary angiography following acute myocardial infarction in the elderly: a matched analysis using propensity scores. J Clin Epidemiol.

[26] Bobrow BJ, Spaite DW, Berg RA, Stolz U, Sanders AB, Kern KB (2010). Chest compression-only CPR by lay rescuers and survival from out-of-hospital cardiac arrest. JAMA.

[27] Kitamura T, Kiyohara K, Nishiyama C, Kiguchi T, Kobayashi D, Kawamura T (2018). Chest compression-only versus conventional cardiopulmonary resuscitation for bystander-witnessed out-of-hospital cardiac arrest of medical origin: A propensity score-matched cohort from 143,500 patients. Resuscitation.

[28] Riva G, Ringh M, Jonsson M, Svensson L, Herlitz J, Claesson A (2019). Survival in out-of-hospital cardiac arrest after standard cardiopulmonary resuscitation or chest compressions only before arrival of emergency medical services: nationwide study during three guideline periods. Circulation.

[29] Borow BJ, Spaite DW, Vadeboncoeur TF, Hu C, Mullins T, Tormala W (2016). Implementation of a regional telephone cardiopulmonary resuscitation program and outcomes after out-of-hospital cardiac arrest. JAMA Cardiol.

[30] Ewy GA, Zuercher M, Hilwig RW, Sanders AB, Berg RA, Otto CW (2007). Improved neurological outcome with continuous chest compressions compared with 30:2 compressions-to-ventilations cardiopulmonary resuscitation in a realistic swine model of out-of-hospital cardiac arrest. Circulation.

[31] Kellermann AL, Hackman BB, Somes G (1989). Dispatcher-assisted cardiopulmonary resuscitation: validation of efficacy. Circulation.

[32] https://clinicaltraials.gov/ct2/show/NCT03981107. Accessed 2 June 2021.

[33] Nishiyama C, Iwami T, Kawamura T, Ando M, Yonemoto N, Hiraide A (2010). Quality of chest compression during continuous CPR: comparison between chest compression-only CPR and conventional CPR. Resuscitation.

[34] Liu S, Vaillancourt C, Kasaboski A, Taljaard M (2016). Bystander fatigue and CPR quality by older bystanders: a randomized crossover trial comparing continuous chest compressions and 30:2 compressions to ventilations. CJEM.

[35] Heidenreich JW, Bonner A, Sanders AB (2012). Rescuer fatigue in the elderly: standard vs. hands-only CPR. J Emerg Med.

[36] López-González A, Sánchez-López M, Garcia-Hermoso A, López-Tendero J, Rabanales-Sotos J, Martínez-Vizcaíno J (2016). Muscular fitness as a mediator of quality cardiopulmonary resuscitation. Am J Emerg Med.

