# Supplementary figures and images for: Dispatcher instructions for bystander cardiopulmonary resuscitation and neurologically intact survival after bystander-witnessed out-of-hospital cardiac arrests: a nationwide, population-based observational study
Source: Crit Care. 2021 Nov 27;25:408. doi: 10.1186/s13054-021-03825-w (PMC8627004; doi:10.1186/s13054-021-03825-w)

## Slide 1
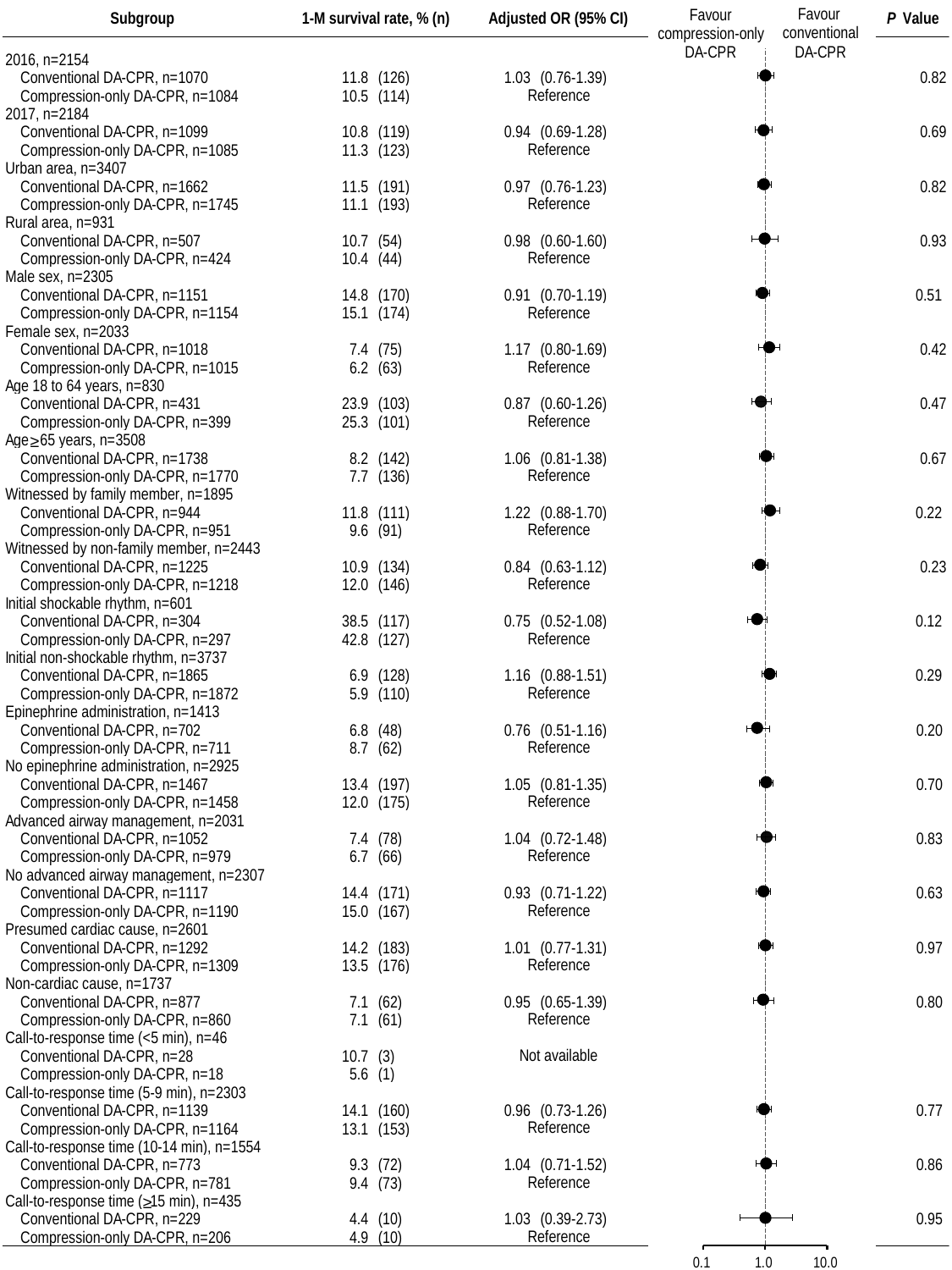

Supplement: Supplementary file 1 — Additional file 1. Figure S1. Subgroup analysis for adjusted odds ratios of conventional DA-CPR for survival rate. CI, confidence interval; DA-CPR, dispatcher-assisted cardiopulmonary resuscitation; OR, odds ratio. A total of 22 subgroup analyses of adjusted ORs of conventional DA-CPR for 1-month survival after propensity score matching compared with compression-only DA-CPR are shown. There were no significant differences in the 1-month survival rate between the two groups. [file 13054_2021_3825_MOESM1_ESM.pptx]

## Slide 1
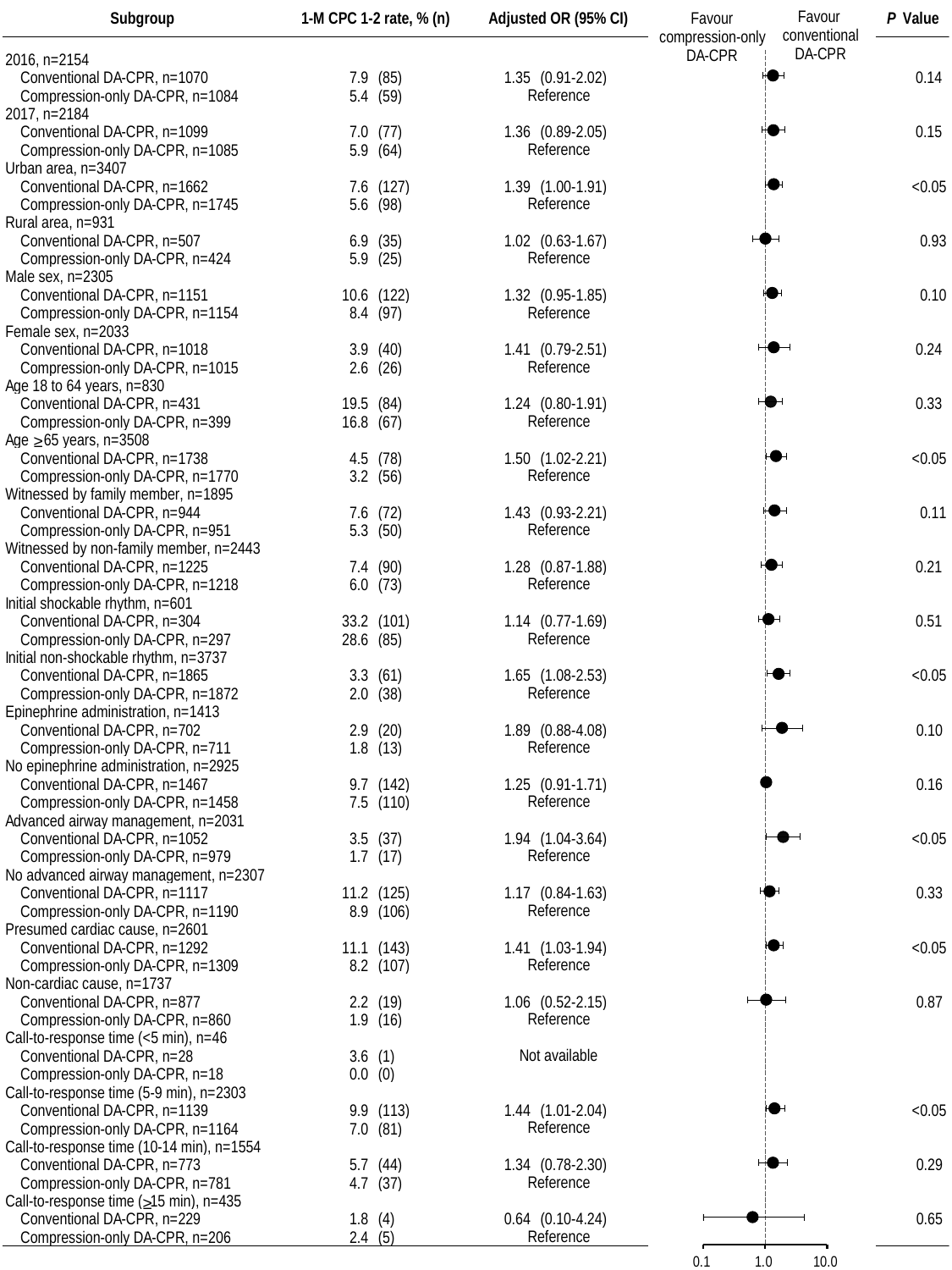

#

Supplement: Supplementary file 2 — Additional file 2. Figure S2. Subgroup analysis for adjusted odds ratios of conventional CPR for CPC 1-2 rate. CI, confidence interval; CPC, cerebral performance category; DA-CPR, dispatcher-assisted cardiopulmonary resuscitation; OR, odds ratio. A total of 22 subgroup analyses of adjusted ORs of conventional DA-CPR for 1-month CPC 1-2 after propensity score matching compared with compression-only DA-CPR are shown. Most subgroup analyses for 1-month CPC 1-2 rate revealed no significant differences between the two groups. However, conventional DA-CPR was associated with increased odds of 1-month CPC 1–2 rate in six subgroup analyses: urban area, age ≥65 years, initial non-shockable rhythm, advanced airway management, presumed cardiac cause, and call-to-response time (5–9 min). [file 13054_2021_3825_MOESM2_ESM.pptx]
